# Efficacy of alpha1-antitrypsin augmentation therapy in conditions other than pulmonary emphysema

**DOI:** 10.1186/1750-1172-6-14

**Published:** 2011-04-12

**Authors:** Ignacio Blanco, Beatriz Lara, Frederick de Serres

**Affiliations:** 1 Biomedical Research Office (OIB-FICYT), Rosal, 7. 33009 Oviedo. Principality of Asturias. Spain; 2 Hospital Universitario Arnau de Vilanova. Avda. Alcalde Rovira Roure 80. 25198. Institut de Recerca Biomédica de Lleida (IRB). Lleida. CIBERES Instituto Salud Carlos III Madrid. Spain; 3 National Institute of Environmental Health Sciences, Research Triangle Park, NC 27709-2233 USA

## Abstract

Up to now alpha 1-antitrypsin (AAT) augmentation therapy has been approved only for commercial use in selected adults with severe AAT deficiency-related pulmonary emphysema (i.e. PI*ZZ genotypes as well as combinations of Z, rare and null alleles expressing AAT serum concentrations <11 μmol/L). However, the compassionate use of augmentation therapy in recent years has proven outstanding efficacy in small cohorts of patients suffering from uncommon AAT deficiency-related diseases other than pulmonary emphysema, such as fibromyalgia, systemic vasculitis, relapsing panniculitis and bronchial asthma. Moreover, a series of preclinical studies provide evidence of the efficacy of AAT augmentation therapy in several infectious diseases, diabetes mellitus and organ transplant rejection. These facts have generated an expanding number of medical applications and patents with claims for other indications of AAT besides pulmonary emphysema. The aim of the present study is to compile and analyze both clinical and histological features of the aforementioned published case studies and reports where AAT augmentation therapy was used for conditions other than pulmonary emphysema. Particularly, our research refers to ten case reports and two clinical trials on AAT augmentation therapy in patients with both AAT deficiency and, at least, one of the following diseases: fibromyalgia, vasculitis, panniculitis and bronchial asthma. In all the cases, AAT was successfully applied whereas previous maximal conventional therapies had failed. In conclusion, laboratory studies in animals and humans as well as larger clinical trials should be, thus, performed in order to determine both the strong clinical efficacy and security of AAT in the treatment of conditions other than pulmonary emphysema.

## Introduction

Alpha1-antitrypsin (AAT) deficiency is an autosomal, codominant genetic disorder, characterized by the polymerization of abnormal, misfolded proteins in the rough endoplasmic reticulum of hepatocytes and, secondarily, a decreased concentration and activity of AAT in blood and tissues. Although several degrees of AAT deficiency are quite common worldwide, severe deficiency (i.e. AAT serum levels below 11 μmol or 50 mg/dL) represents a rare condition affecting 1 per 2,000-5,000 Caucasian individuals of Western European origin, with about a quarter or half of them developing clinically significant diseases [1,2].

Human alpha-1 antitrypsin (AAT), also called alpha-1 proteinase inhibitor (PI), and SERPINA1 (Serine Protease Inhibitor, clade A, member 1), is a 394-amino acid, circulating glycoprotein with a molecular weigh of 52-kDa. AAT is medium sized and globular in shape (6.7 × 3.2 nm), water soluble and diffusible into different tissues, with an average of 4-5 day life span in circulating blood. AAT is mainly secreted by hepatocytes (~80%) and, to a minor extent, by monocytes, pancreatic islets, lung alveolar cells and colonic enterocytes, reaching high mean concentrations in plasma (1-2 g/L). Eighty percent of plasmatic protein diffuses into different body tissues and approximately 10% reaches most biological fluids [3].

Extensive data show that the major function of AAT is the inhibition of over-expressed neutrophil elastase and other serine proteinases (i.e. proteinase-3, myeloperoxidase and cathepsin G from neutrophils, tryptase and chymase from mast cells, etc.) in order to maintain the physiological proteinase-antiproteinase balance [4-34]. Moreover, an increasing number of new inhibitory and non-inhibitory properties conferring to ATT outstanding anti-inflammatory, inmunomodulatory and antimicrobial attributes have been discovered in later years, together with the fact that AAT activity is not only limited to lungs but also to most body tissues [35-38].

AAT gene has two alleles. Normal alleles are called M; thus, normal individuals have genotype MM. The most common severe deficiency allele is called Z (Glu342Lys); in clinical practice over 95% of severely deficiency patients have genotype ZZ (expressing 10-20% of serum AAT). The remaining 5% belongs to patients with genotype SZ and about 30 rare deficiency and null phenotypes, expressing 0-30% AAT serum levels. Most common diseases related to severe AAT-deficiency are pulmonary emphysema (found in around 3% of adults with COPD), liver cirrhosis (2.5% of children and 10-20% of PI*Z adult homozygotes), systemic vasculitis (7%) and relapsing panniculitis (0.1%) [3,39,40].

In 1987 the Food and Drug Administration (FDA) published the first guidelines on augmentation therapy with intravenous infusions of purified AAT obtained from human serum of adults suffering from moderate pulmonary emphysema (FEV1 30-65%) and severe AAT deficiency (i.e. PI*ZZ genotypes and combinations of Z, rare and null alleles expressing AAT serum concentrations < 11 μmol or 50 mg/dL) [41]. Taking into account scientific evidence and knowledge, the American Thoracic Society, the European Respiratory Society, the American College of Chest Physicians and the American Association for Respiratory Care recommend augmentation therapy for selected patients with lung emphysema. Augmentation therapy not only has been approved in the US but also in Germany, Canada, Italy, Spain, Austria, Ukraine, Argentina and Brazil, with similar criteria of use [3,42-52].

In the last years, preclinical studies in cells, animal models and small cohorts of humans provided initial evidence for the efficacy of AAT in several infectious diseases, diabetes mellitus, organ transplant rejection and other possible inflammatory diseases [38,53-60]. As a consequence, the number of medical applications and patents for other indications of AAT other than pulmonary emphysema is increasing http://www.omnibiopharma.com/development. In this context, clinical observations describing the remarkable (even impressive) efficacy of the augmentation therapy in AAT deficiency patients with fibromyalgia, systemic vasculitis, panniculitis and bronchial asthma have also been reported [61-70]. According to Jenicek's statement on clinical case reporting, these observations should be taken into account: *"Although a case report has the weakest level of Evidence Based Medicine, sometimes an unusual case may become an "index case", and it may lead to the formulation of new hypothesis on diagnostic and therapeutic options" *[71]. The aim of the present manuscript is to compile published case reports and small trials where augmentation therapy was provided, on a compassionate basis, to humans with disorders other than pulmonary emphysema and to discuss their results in the light of current knowledge on AAT deficiency-related disorders.

## Methods

Firstly, a research on the MEDLINE database (1966-2010) was made using the generic terms: "alpha-1 antitrypsin", "alpha-1-proteinase inhibitor" and "alpha-1 antitrypsin deficiency" and combining them with one of the following key concepts: "augmentation therapy", "replacement therapy", "treatment". Afterwards, a more specific search was carried out adding to the previous terms one of the following terms: "infectious diseases", "inflammatory diseases", "associated diseases", ("NOT emphysema"), "fibromyalgia", "human immunodeficiency virus", "HIV", panniculitis", "vasculitis", "bronchial asthma", "graft rejection", "diabetes", "influenza", "tuberculosis" and "anthrax". We also consulted EMBASE Excerpta Medica, SciVerse Scopus and the Cochrane Library databases in order to search for abstracts of similar types of papers, as well as bibliographic references of previously retrieved articles, authors' databases and reviews from several experts on this topic.

Only reports related to human beings were selected. Studies on augmentation therapy for pulmonary emphysema have not been included in the present analysis, considering this topic to be out of the scope of the present manuscript.

## Results

Ten case reports [61-68] and two clinical trials [69,70] have been found on intravenous AAT augmentation therapy in 12 patients with both AAT deficiency and one of the following diseases: vasculitis, panniculitis, fibromyalgia and bronchial asthma. Ten of these 12 treated patients had genotype PI*ZZ [61-68] and the remaining 2, PI*MZ.[69,70].

### 1. Vasculitis

**Figure 1:**
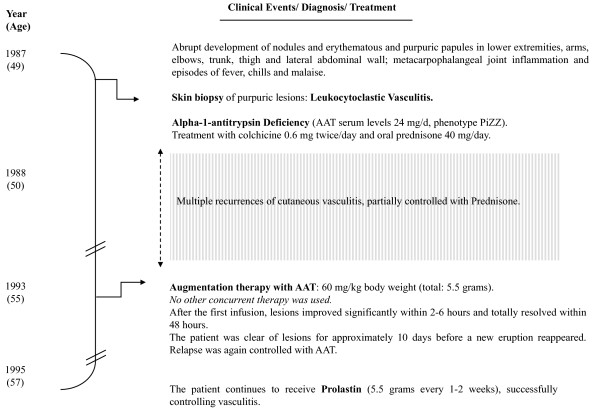
**Timeline of clinical events in a case of leukocytoclastic vasculitis related to ZZ alpha-1-antitrypsin deficiency**. AAT: alpha-1-antitrypsin.

In 1995 Dowd et al (University of Louisville School of Medicine, Louisville, Kentucky) reported a case of to "a 49-year-old man, with a PI*ZZ genotype and AAT serum of 24 mg/d, suffering from extensive cutaneous leukocytoclastic vasculitis, with multiple recurrences partially controlled with colchicine, prednisone, plasma infusions and plasma-exchange therapy" [61]. Authors describe the occurrence of repetitive outbreaks of fever, chills, purpuric papules and nodules in lower extremities, arms, face, trunk, hands and the lateral abdominal wall, provoking large vitiligo areas in elbows, knees, dorsal hand and pretibial surfaces. A biopsy skin sample showed alterations related to leukocytoclastic vasculitis (i.e. inflammatory involvement of medium and small vessels, red blood cell extravasations, edema and clear neutrophilic invasion with leukocytoclasia (disrupted neutrophils). The results of a complete blood cell count, serum multiphasic analysis and urinalysis were normal. Studies with normal or negative findings included assays for antinuclear antibody, antibodies to extractable nuclear antigen, antinuclear DNA, complement levels and hepatitis B surface antigen. The chest roentgenogram was within normal limits as well as pulmonary function tests. Five years later, the patient displayed a "dramatic response after 1 dose of Prolastin^® ^(60/mg/Kg), with improvement <6 hrs, and total resolution <48 hrs" and "good control with long-term augmentation therapy (60/mg/Kg)/1-2 weeks" (Figure 1).

### 2. Panniculitis

**Figure 2:**
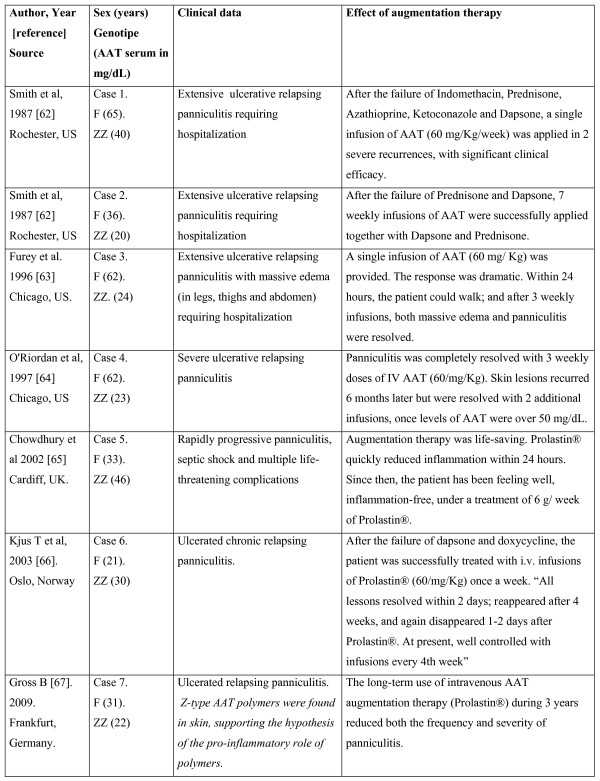
**Demographic and clinical data of 7 patients with relapsing panniculitis associated to ZZ alpha-1-antitrypsin deficiency treated with alpha-1-antitrypsin augmentation therapy**. F: female. AAT: alpha-1-antitrypsin.

From 1987 to 2010 seven cases of AAT deficiency-related refractory relapsing panniculitis, treated with AAT as the last resource, were published [62-67]. The two first cases were reported by clinicians in the Mayo Clinic, Rochester, Minn, in 1987 [62]. Ten years later, 2 other cases were reported by the Northwestern University Medical School of Chicago [63,64], and, from 2002, 3 new cases (1 each from the UK [65], Norway [66] and Germany [67]) were reported by several European authors. All these 7 patients were females with genotypes PI*ZZ. Their mean AAT serum concentrations were 29 mg/dL (range: 20-46), and their mean age 44 years (range: 21-65). In all the cases sequential infusions of AAT were applied after the repetitive failure of conventional therapies [i.e. antibiotics (doxycycline, minocycline, cloxacillin and nafcilina), dapsone, systemic corticosteroids, ciclofosfamida, chloroquine, nitrogen mustard, plasma infusions, plasmapheresis, etceteras]. As a whole, skin samples showed a high expression of cell components, being neutrophil invasion the most relevant finding. They also disclosed the variable presence of scattered monocytes, lymphocytes and macrophages, areas of tissue destruction, fibroblasts and fibrosis, inflammation and rupture of fibrous septa between fat lobules, liquefactive necrosis, elastin breakdown, scars, fistulas and ulcers. In the only case where Z polymers were investigated, they were demonstrated to appear both in active lesions and surrounding non-inflamed tissues [67]. The response to AAT infusions was uniformly positive in all the cases, being described in some reports as "dramatic", "very-high and rapid", "life-saving", etc. (Figure 2).

In the same context, the results of two liver transplants in PI*ZZ patients with end-stage liver cirrhosis and AAT deficiency-related panniculitis will be summarized. In both cases, a rapid and steady control of panniculitis was observed after the replacement of each damaged liver by a new efficient organ, restoring AAT serum levels back to normal. The first case was reported in 1997 by O'Riordan et al. (Northwestern University Medical School, Chicago, IL, USA) [64]. Authors observed evolution in "a 53-year old man...ZZ (AAT: 17 mg/100 ml)...with end-stage liver cirrhosis and relapsing panniculitis... [who] received a liver transplant. His new phenotype was PI*MM... and serum AAT of 153 mg/dL. One month after...a complete resolution of the skin lesions" was noticed.

The second case was reported by Fernandez-Torre et al. from the Hospital Juan Canalejo, La Coruña, Spain, in 2009 [72]. On the contrary, authors noticed alterations in "A 56-year-old MM patient [who] acquired a ZZ phenotype after a liver transplant for alcoholic liver cirrhosis. Subsequently, he developed cirrhosis and panniculitis. Liver transplantation was again performed, with complete resolution of the skin lesions and restoration of AAT serum levels (from 28 to 270 mg/dl)...11 months later he remains asymptomatic".

In the same context, de Oliveira et al. (São Paulo, Brazil) published in 2004 the case of a 23-year-old patient with extensive, ulcerative relapsing panniculitis, and AAT serum levels of 22 mg/dL and phenotype ZZ [73]. The patient was treated with 16 sessions of plasma exchange therapy, with cutaneous lesions clinically controlled after five sessions. AAT increased to 60 mg/dL after the first session, slowly decreasing to initial levels in approximately 15 days until the next plasma infusion (when AAT levels increased again and, thus, relapses were promptly controlled). This case shows many similarities with the first report on the use of plasma exchange therapy in panniculitis, made in 1986 by Viraben et al. [74], who used fresh plasma every other day for 2 weeks, with dramatic effectiveness. Both authors consider plasma exchange as an effective therapeutic alternative for unresponsive patients to conventional treatments, thus highlighting the necessity of further studies with a larger number of individuals.

### 3. Fibromyalgia

Up to date three cases have been reported by Blanco et al. (Hospital Valle del Nalón, Principality of Asturias, Spain) [68,69].

**Figure 3:**
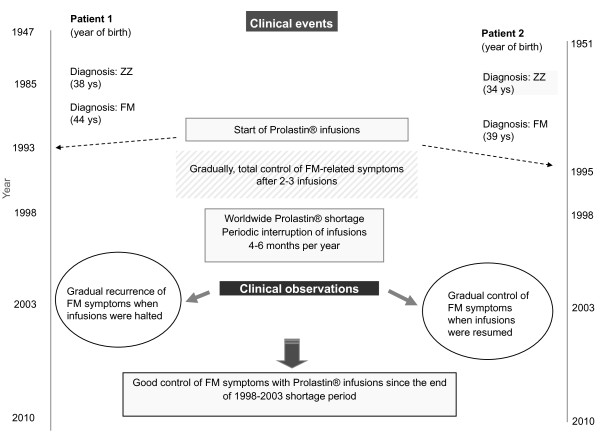
**Effects of augmentation therapy in 2 ZZ Alpha-1-antitrypsin deficiency sisters with fibromyalgia (FM)**.

**Cases 1 and 2 **were reported in 2004 [68]. In the early nineties two PI*ZZ Spanish sisters with severe fibromyalgia started AAT replacement therapy. During the next 3 and 5 years, respectively, they both experienced a rapid, progressive and constant control of fibromyalgia symptoms. However, a commercial shortage of AAT by 1998 led to the annual interruption of infusions for 4-6 consecutive months during 5 years. As a result, fibromyalgia symptoms recurred during infusion interruption and disappeared completely whenever infusions were resumed. Currently, both patients are regularly treated with Prolastin^®^, and do not have fibromyalgia symptoms anymore (Figure 3).

**Figure 4:**
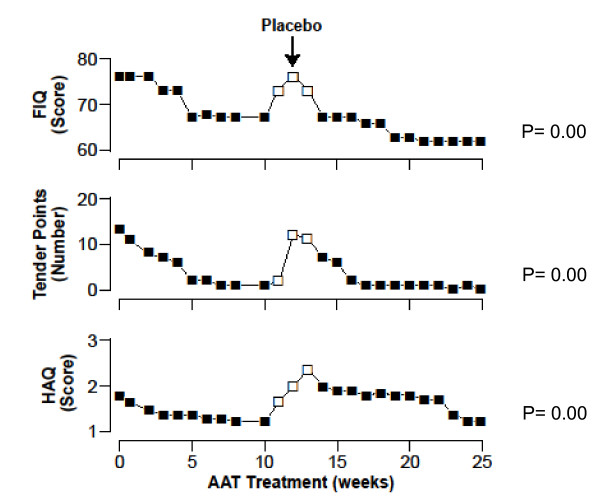
**Effects of a 6-month trial with alpha-1-antitrypsin augmentation therapy and placebo in a Fibromyalgia and Chronic Fatigue Syndrome patient with intermediate MZ alpha-1-antitrypsin deficiency**. FIQ: Fibromyalgia Impact Questionnaire. HAQ: Health Assessment Questionnaire.

**Case 3 **was reported in 2010 [69]. In 2004 a patient with both AAT deficiency (MZ) and severe fibromyalgia/CFS (Chronic Fatigue Syndrome) participated in a trial with AAT-intravenous augmentation therapy after the failure of conventional therapies. Since then, the patient has experienced a very good clinical response (Figure 4).

At present, the 3 patients continue to be under long-term AAT augmentation therapy with successful results.

### 4. Bronchial asthma

**Figure 5:**
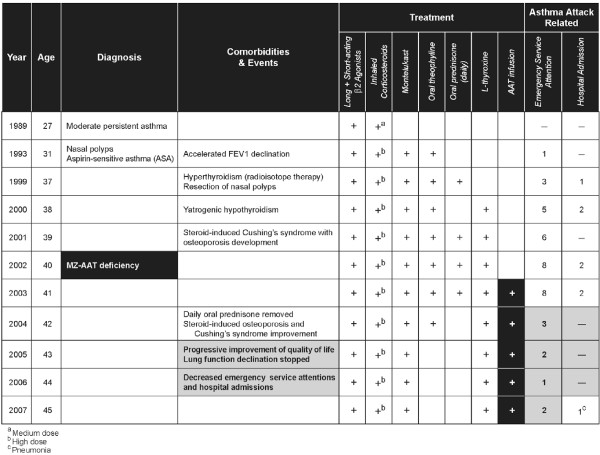
**Chronology of clinical events in an MZ-AAT patient with severe persistent asthma**. AAT: Alpha1-antitrypsin. FEV1: Forced Expiratory Volume in 1 second.

**Figure 6:**
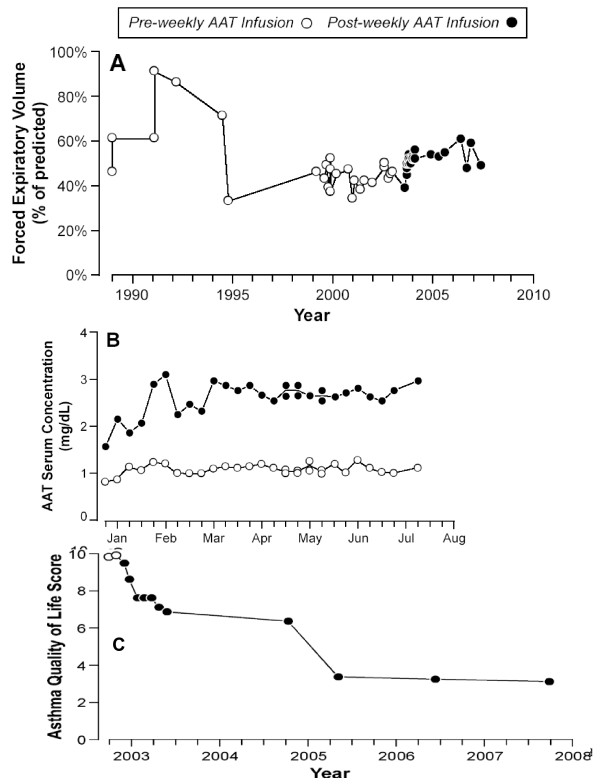
**Effects of long-term augmentation therapy with alpha-1 antitrypsin in an MZ-AAT severe persistent asthma**. Forced Expiratory Volume (A), Alpha-1-antitrypsin (AAT) serum concentrations (B), and Asthma Quality of Life (C) scores after and before alpha-1-antitrypsin infusions.

In 2008, Blanco I, et al. (Hospital Valle del Nalón, Asturias, Spain) reported the case of a Caucasian 27-year old woman, working as a cleaner, who started to suffer from recurrent dyspnoea, wheezing, cough and chest tightness [70]. A totally reversible and variable spirometric airflow limitation was evidenced, and she was diagnosed with bronchial asthma (Figure 5). In the following years, she was also diagnosed with nasal polyps as well as Aspirin-Sensitive Asthma (ASA). Isoelectric-focusing and nephelometry showed that the patient had a deficient phenotype MZ with intermediate low AAT serum concentrations. On the other hand, PCR genotyping sequenced exons II-V of the AAT gene, diagnosing a genotype M_3_Z. The results of several studies to rule out other conditions, mimicking or worsening bronchial asthma, were either normal or negative except for immune hyperthyroidism. The patient was firstly treated with Carbimazole and later with radioisotope therapy (causing iatrogenic hypothyroidism but easily managed with Levothyroxine). However, asthma persisted with frequent at night symptoms, daily attacks of dyspnoea and limited physical exertion. As pulmonary function significantly worsened due to asthma attacks, the patient had to use frequently the emergency medical services and even was admitted to hospital in several occasions. She was prescribed long-term prednisone tablets (15-30 mg/day). However, asthma continued to be out of control and bronchial obstruction, permanent. Moreover, significant side effects of glucocorticoid drugs appeared in the following months. As a consequence, the patient experienced severe limitation of her physical capacity which negatively affected her mental state and social and personal life. The results of a computerized axial tomography of the chest to rule out pulmonary emphysema and other intercurrent complications were within normal limits. Due to the unfavourable clinical evolution of the patient, doctors tried a therapeutic trial with AAT augmentation therapy (60 mg/Kg/week dose for the first two weeks and 120 mg/biweekly for 7 months). As the results of the trial were significantly favourable, the treatment continued. The continuous administration of AAT infusions stopped the decline in lung function. In 1991-1994 (period 1) her FEV1 (expressed by mean and standard deviation) was equivalent to 69% (17); in 1995-2003 (period 2) it declined to 43% (5.14); and from 2004, once the AAT augmentation therapy started, to 2007 (period 3), it rose up to 52% (3.75). Statistical significance was found between period 1 and period 2 (p = 0.000), period 1 and period 3 (p = 0.000), and period 2 and period 3 (p = 0.001). As expected, the augmentation therapy increased the AAT serum levels of the patient, measured by nephelometry and expressed in mg/dL, from basal values of 0.78 (0.04) to 1.08 (0.10) pre-infusions and 2.61 (0.34) post-infusions. Moreover, AAT augmentation therapy significantly reduced the number of emergency visits and hospital admissions (22 emergency visits in 2001-2003 vs. 8 in 2004-2007; and 4 hospital admissions in 2002-2003 vs. 1 in 2004-2007). The need for prednisone was also reduced shortly after the beginning of the augmentation therapy, facilitating the elimination of chronic corticoid side effects and progressively reducing the high score of Asthma Quality of Life (AQL) in 2003 from 10 to 3 points in 2007 (Figure 6).

## Conclusions

AAT deficiency is a congenital disorder with a remarkable variability in its clinical presentation [3]: some subjects can develop liver cirrhosis and others pulmonary emphysema; to a minor extent, they can also develop panniculitis, vasculitis, bronchial asthma or fibromyalgia; and more than one third of the remaining carriers can stay symptomless their entire life.

The 12 subjects mentioned in the present paper suffered from less common AAT deficiency-related diseases (i.e. leukocitoclastic vasculitis; relapsing panniculitis, fibromyalgia, and refractory asthma). All these patients have in common AAT deficiency genotypes (10 ZZ, 2 MZ). Also they all experienced a positive response to intravenous AAT augmentation therapy, applied on a compassionate basis as the last resource after the repetitive failure of conventional therapies and the extremely difficult clinical control of AAT deficiency-related diseases (life-threatening in some cases).

The skin samples of the patient with leukocytoclastic vasculitis related to ZZ AAT deficiency showed acute and chronic inflammation. Neither anti-neutrophil cytoplasmic antibodies (ANCA) nor anti-neutrophil enzyme proteinase 3 (PR3-ANCA) antibodies were analyzed. Although the pathophysiological mechanisms of vasculitis have not been determined yet, they seem to be related to inflammatory mechanisms mediated by neutrophil invasion of the small and medium vessels. The excess of proteinase-3, not neutralized by AAT, could also play an important role in AAT deficiency subjects with antineutrophilic cytoplasmatic antibody C-ANCA positive vasculitis. Since AAT is a major inhibitor of PR3, the PR3-AAT imbalance in AAT deficiency-related vasculitis could lead to increased circulating levels of PR3 and trigger the synthesis of PR3-ANCA antibodies [39]. AAT deficiency-related vasculitis would be, therefore, restricted to PR3-ANCA-positive cases and not to less common occurrences of MPO-positive or ANCA-negative cases. On the other hand, patients with both vasculitis and AAT deficiency have been reported to have a reduced ability to bind PR3 released by neutrophils previously activated, thus promoting PR3-mediated proteolytic vessel damage. Actually, several findings show that the disease course of Wegener's granulomatosis (WG) is more severe when it is related to AAT deficiency than when it is not. This supports the hypothesis that AAT deficiency could trigger vasculitis by means of PR3-ANCA autoimmunity [40]. The role of circulating Z polymers, attacking the endothelial walls of blood vessels and promoting vasculitic responses, has not been determined yet nor whether all patients with manifestations of vasculitis and severe AAT deficiency could benefit from AAT augmentation therapy. However, the administration of this glycoprotein seems to be logical for selected cases of AAT deficiency-related refractory vasculitis.

The pathophysiological mechanisms of panniculitis in AAT deficiency subjects have not been determined yet. As some hypotheses suggest an inflammatory process mediated by neutrophils invading the subcutaneous fat layer, the most logical mechanism seems to be the unopposed anti-inflammatory proteolytic damage by neutrophil-serine proteinases in the context of deficient serum levels of AAT [39]. In one of the aforementioned cases of the present study, Z polymers were found in both active lesions and surrounding non-inflamed tissues, suggesting a potential pro-inflammatory role of polymers in the development of panniculitis. The histological features of panniculitis, especially neutrophil invasion, could explain the rapid clinical resolution of inflammation with AAT augmentation therapy. Therefore, its application should be tried in cases which fail to respond to conventional therapeutic approaches.

Fibromyalgia syndrome is characterized by the central amplification of sensory impulses (central sensitization) causing autonomic, hormonal, immune and cytokine interactions and perturbations. Fibromyalgia can result from the disordered expression of inflammatory substances, amplified by genetic factors. Recent evidence indicates that fibromyalgia can be added to the list of AAT deficiency-related diseases. Main evidence elements include: **(I) **The short and long-term repetitive positive response to AAT augmentation therapy showed by the three aforementioned patients with both AAT deficiency and fibromyalgia [68,69]. **(II) **An epidemiological study showing a twofold higher frequency of PI*Z allele in fibromyalgia patients than in general population (4% vs. 2%), with a calculated prevalence of MZ, SZ and ZZ deficiency genotypes being 2-4 times higher in fibromyalgia patients than in general population [75]. Therefore, AAT deficiency should be considered as a predisposing factor for the development of early and severe onset of fibromyalgia in a subgroup of patients with AAT serum levels lower than normal (7% MZ, 0.5% SZ and 0.2% ZZ). **(III) **A case-control study showing plasma levels of MCP-1, VEGF and TNF-alpha significantly lower in AAT deficiency subjects with fibromyalgia than in controls [76]. **(IV) **A blinded inmunohistochemical study of skin biopsies performed in 112 subjects (63 fibromyalgia vs. 49 controls) with AAT normal and deficient genotypes. The results showed a significantly increased number of mast cells in the dermis of fibromyalgia patients, with a proportion fibromyalgia/controls of about 5:1 cells per microscopic high power field (p = 0.000) [77]. Mastocytes positively stained with tryptase, AAT and PAR-2 antibodies as well as with a kit receptor marker (CD117) and a cell activation marker (CD63), showing that about 50-80% of these mastocytes were activated. Remarkably, the skin samples of the 3 patients under long-term AAT augmentation therapy did not show any clinical fibromyalgia manifestations but also an increased number of dermal mast cells. Therefore, exogenous AAT could serve as a mast cell stabiliser and, as demonstrated in some laboratory studies, as a neutraliser of certain mast cells mediators (i.e. histamine and serine proteinases, such as tryptase and chymase) [17-19]. AAT augmentation therapy should be considered to treat selected cases of AAT deficiency-related refractory fibromyalgia not responding to conventional therapeutic measures, and larger trials are needed to determine the strong clinical efficacy of AAT in fibromyalgia patients.

Histological samples from airways were not collected in the case of the patient with bronchial asthma. However, it is worth noting that severe asthma is considered as an inflammatory airway disease related to sub-acute and chronic inflammation maintained by eosinophils, mastocytes, dendritic, epithelial and endothelial cells, basophiles, T lymphocytes, macrophages and neutrophils. Therefore, AAT could also play a determining anti-inflammatory role in counteracting the effects of released oxidants, cytokines, proteinases and other inflammation mediators. Precisely, the imbalance between elastase and AAT in asthmatic subjects has been already described as well as the relation of airway inflammation to abnormal high levels of active elastase, detected in patients' samples of induced sputum [78]. Neutrophil elastase has not only proteolytic properties but also acts as a powerful secretagogue. Particularly, recent evidence indicates the persistence of neutrophilic and eosinophilic inflammation in patients' airways with severe asthma, and the activation by neutrophil proteinases (i.e. elastase, cathepsin G and proteinase-3) of peripheral blood leukocytes in order to produce superoxide and proinflammatory cytokines, which can further aggravate airway inflammation [79]. On the other hand, it is worth mentioning that the airway smooth muscle (ASM) not only has contractile properties but also is involved in the pathogenesis of asthma, producing inflammatory mediators. Human neutrophil elastase is a mitogen which activates ASM cells through extracellular signal-regulated kinase (ERK) signaling pathway [80]. Other interesting study shows that the allergic stimulation of airways provokes the release of elastase, tissue kallikrein (TK) and reactive oxygen species (ROS), reducing AAT activity and contributing to airway hyperreactivity (AHR) [81]. Moreover, the bronchoconstriction caused by elastase, high-molecular-weight kininogen and ROS and AHR induced by both ROS and antigen was stopped thanks to the application of 10 mg of recombinant AAT, suggesting the key role of AAT in regulating airway responsiveness [82]. In conclusion, the positive effects of AAT augmentation therapy observed in this case support the hypothesis of a proteinase-antiproteinase imbalance in the pathogenesis of asthma, reaffirming the need for further studies on the mechanisms of this bronchial disease and the efficacy of AAT augmentation therapy in selected AAT deficiency subjects with refractory asthma.

In summary, all these 12 cases should promote further laboratory studies in animals and humans as well as larger clinical trials in order to determine the strong efficacy of AAT augmentation therapy in diseases other than pulmonary emphysema, especially as a powerful anti-inflammatory agent in processes such as fibromyalgia, vasculitis, panniculitis and bronchial asthma.

## Competing interests

The authors declare that they have no competing interests.

## Authors' contributions

IB conceived of the study, participated in its design and coordination, carried out data collection and analysis and drafted the manuscript. FS and BL carried out data collection and participated in the design and analysis of the study. All authors read and approved the final manuscript.
